# Intelligence Quotient changes over 10 years: diversity of cognitive profiles in first episode of psychosis and healthy controls

**DOI:** 10.1192/j.eurpsy.2023.1311

**Published:** 2023-07-19

**Authors:** N. Murillo-Garcia, V. Ortíz-García de la Foz, M. Miguel-Corredera, E. Setién-Suero, K. Neergaard, J. Moya-Higueras, B. Crespo-Facorro, R. Ayesa-Arriola

**Affiliations:** 1 Research Institute Marqués de Valdecilla (IDIVAL), Santander; 2 University of Lleida, Lleida; 3 CIBERSAM, Madrid, Spain

## Abstract

**Introduction:**

The evidence on the course of the intelligence quotient (IQ) at the long term in individuals with schizophrenia spectrums disorders is inconclusive.

**Objectives:**

We aimed to analyse whether IQ improves, declines, or remains stable over 10 years in a sample of patients with First Episode Psychosis (FEP) and healthy controls (HCs).

**Methods:**

The FEP patients participated in a Program of First Episode Psychosis in Spain called PAFIP. At baseline, FEP patients provided demographic and clinical data, and completed a neuropsychological assessment that included an estimation of premorbid IQ trough the WAIS vocabulary subtest. At 10-year follow-up, the participants were invited to complete the same evaluation and 10-year IQ was estimated. The group of HCs underwent the same neuropsychological battery at both moments. Cluster analysis was performed separately in the FEP patients and the HCs to determine their profiles of intellectual change.

**Results:**

FEP patients (n=137) were grouped into five clusters (see Figure 1): “Improved low IQ” (9.49% of patients), “Improved average IQ” (14.6%), “Preserved low IQ” (17.52%), “Preserved average IQ” (43.06%), and “Preserved high IQ” (15.33%). Ninety HCs were grouped into three clusters: “Preserved low IQ” (32.22% of the HC), “Preserved average IQ” (44.44%), and “Preserved high IQ” (23.33%). Demographic data of FEP patients are presented in Table 1.

**Table 1.** Sociodemographic data of FEP patients

	**Improved low IQ**	**Improved average IQ**	**Preserved low IQ**	**Preserved average IQ**	**Preserved high IQ**		
	(C1)	(C2)	(C3)	(C4)	(C5)		
	N= 13	N= 20	N= 24	N= 59	N= 21		
	Mean (SD)	Mean (SD)	Mean (SD)	Mean (SD)	Mean (SD)	F	P
Premorbid IQ	71.15 (6.50)	84.50 (5.10)	88.96 (5.31)	100.76 (4.90)	117.14 (7.34)	180.87	<0.001
10-year IQ	85.38 (5.94)	103.25 (4.06)	90.00 (5.32)	105.76 (6.49)	114.52 (6.87)	77.47	<0.001
Age	26.44 (6.07)	24.85 (4.08)	25.99 (8.49)	30.86 (9.54)	33.20 (8.81)	4.350	0.002
Age of onset	25.54 (5.81)	24.11 (4.19)	25.46 (8.41)	29.68 (9.26)	32.14 (8.48)	3.993	0.004
Sex (male %)	53.85	80.00	62.50	49.15	42.86	X= 7.672	0.104
Years of education	8.31 (2.14)	9.00 (2.10)	9.00 (2.13)	11.63 (3.39)	14.38 (3.15)	15.818	<0.001
DUP (months)	10.77 (16.50)	8.94 (9.79)	6.42 (9.47)	14.08 (28.46)	12.77 (20.02)	0.628	0.643
Schizophrenia diagnosis (yes%)	53.84	70.00	70.83	59.32	57.14	2.096	0.718

**Conclusions:**

The FEP patients showed intellectual improvement or stability, but no decline post-onset of psychosis. However, their profiles of intellectual change are more heterogeneous than that of HCs over 10 years. Particularly, there is a subgroup of FEP patients with a significant potential for long-term cognitive enhancement.

**Disclosure of Interest:**

None Declared

